# Nano-Engineered Sandwich Interlayers for Simultaneous Functionalization and Delamination Resistance in CFRPs

**DOI:** 10.3390/polym18080957

**Published:** 2026-04-14

**Authors:** Pengzhe Ji, Yunxiao Zhang, Yunfu Ou, Juan Li, Dongsheng Mao

**Affiliations:** 1 School of Materials Science and Chemical Engineering, Ningbo University, Ningbo 315211, China; 2 State Key Laboratory of Advanced Marine Materials, Zhejiang Key Laboratory of Extreme-environmental Material Surfaces and Interfaces, Ningbo Institute of Materials Technology and Engineering, Chinese Academy of Sciences, Ningbo 315201, China; 3 School of Chemical Sciences, University of Chinese Academy of Sciences, Beijing 100049, China; 4 School of Materials Science and Engineering, NingboTech University, Ningbo 315100, China

**Keywords:** carbon fiber-reinforced polymers, carbon nanotubes, sandwich-structured interlayers, glass fiber mesh fabric, Mode II interlaminar fracture toughness, interlaminar delamination

## Abstract

Carbon fiber-reinforced polymers (CFRP) are widely employed in advanced manufacturing sectors such as aerospace, wind energy, and new energy vehicles owing to their high specific strength and stiffness. The growing demand for lightweight, high-performance, and multifunctional materials has accelerated the development of structurally and functionally integrated CFRP. Introducing functional interlayers between composite laminates is an effective strategy to impart additional functionalities; however, such interlayers are often multi-component and structurally complex. A critical challenge remains to integrate functionality without compromising, and preferably enhancing, the load-bearing capability of CFRP, particularly their resistance to interlaminar delamination. In this study, electrically heated CFRP incorporating a sandwich-structured interlayer composed of glass fiber mesh fabric/CNT veils doped with carbon nanotubes/glass fiber mesh fabric (GF/CNTs-CNTv/GF) was investigated. The effects of interlayer architecture and CNT loading on the Mode II interlaminar fracture toughness were systematically examined. Delamination failure modes and interlaminar toughening mechanisms were analyzed using scanning electron microscopy and ultra-depth-of-field three-dimensional microscopy. The results demonstrate that an optimal CNT pre-impregnation concentration of 1.0 wt% yielded a maximum G_IIC_ of 1644.8 J/m^2^, corresponding to a 103.06% increase relative to the reference laminate. The enhanced performance is attributed to simultaneous optimization of interfacial “nano-engineering” effects, including matrix toughening and a pronounced “nano-anchoring” mechanism induced by CNT. These effects promote a transition in failure mode from weak interfacial debonding to a mesh-block composite delamination pattern, thereby activating multiple energy-dissipation mechanisms such as crack deflection, fiber pull-out, rupture, and bridging. This work highlights the effectiveness of CNT-modified sandwich interlayers in improving delamination resistance and provides both theoretical insight and experimental validation for the design of multifunctional CFRP with superior interlaminar fracture toughness.

## 1. Introduction

Carbon fiber-reinforced polymers are esteemed as premier materials for primary load-bearing structures due to their remarkable high specific strength and stiffness, excellent fatigue resistance, and outstanding design flexibility. These composites have been extensively utilized in advanced manufacturing sectors, including aerospace, wind energy, new energy vehicles, and high-end sports equipment [1,2]. The escalating engineering demand for lightweight, high-performance, and multifunctional materials is rapidly outpacing the capabilities of traditional monolithic materials, particularly under extreme in-service conditions like those in high-altitude, polar, and alpine environments. This has catalyzed the development of structural–functional integrated materials, which incorporate additional functions such as sensing, energy management, and environmental adaptability, alongside load-bearing capacity. These materials significantly enhance the overall performance and reliability of systems while efficiently managing weight and structural complexity [3,4]. Beyond these traditional advanced manufacturing applications, fiber-reinforced polymer (FRP) sandwich structures have also shown growing potential in civil engineering because of their low self-weight, high specific mechanical properties, corrosion resistance, and design flexibility. Recent studies have highlighted their suitability for bridge deck systems, building floor components, and other lightweight load-bearing structural elements [5]. For such applications, interlaminar damage tolerance is of particular importance, since delamination can severely compromise structural integrity and long-term service reliability. Therefore, improving the interlaminar fracture resistance of FRP sandwich structures is of great significance not only for advanced multifunctional composites but also for their broader implementation in civil engineering.

The incorporation of embedded functional interlayers into the CFRP interlaminar region constitutes an effective strategy for the structural–functional integrated design of such materials. For instance, surface ice accretion poses a serious threat to the operational safety and efficiency of equipment in cold environments, such as aircraft and wind turbines [6]. By integrating electro-thermal functional elements as interior interlayers within CFRP, researchers have enabled efficient, low-energy, and active surface heating and de-icing [7]. Yao et al. [8] fabricated a glass fiber (GF) composite electrothermal structure using a highly aligned carbon nanotube web, which effectively removed ice accumulation in the heating area within 15 s at −12 °C under a heat flux of 4.9 kW/m^2^. Guo et al. [9] developed a composite electrothermal structure based on an ultra-thin flexible electrothermal heating film, achieving complete melting of the ice layer interface in the heating zone within 50 s at −9 °C under a heat flux of 9 kW/m^2^. Notably, due to the excellent intrinsic electrical conductivity of carbon fiber reinforcements [10], the direct embedding of electrothermal functional layers into composites is susceptible to internal short circuits, which diminishes energy utilization efficiency and poses significant safety hazards. Consequently, electrothermal interlayer components embedded in CFRP must be designed with insulating protective layers, typically employing a sandwich structure of insulation layer/functional layer/insulation layer to ensure circuit isolation and protection.

Consequently, such electrothermal interlayers are multicomponent, structurally complex systems. A core design challenge for interleaved composites is thus to integrate functions while preserving or even improving critical load-bearing properties, particularly delamination resistance. However, existing research has predominantly concentrated on the realization and optimization of heating functions, overlooking the potential adverse effects of functional element integration on the interlaminar mechanical properties of CFRP. Currently, electrothermal elements commonly used can be broadly classified into three categories: traditional metal-based conductive materials [11] (e.g., nickel-chromium alloys, iron-chromium-aluminum alloys, and copper-based alloys), carbon nanomaterial-based electrothermal elements [12] (such as carbon nanotube films and graphene coatings), and conductive polymer-based materials [13] (including polyaniline- and polypyrrole-based composite layers). Among these, traditional metal-based electrothermal elements can provide high heating efficiency; however, their intrinsic high stiffness and pronounced modulus mismatch with polymer resin matrices result in weak interfacial bonding strength [14]. This mismatch readily induces stress concentration in the interlaminar region under thermal or mechanical loading, thereby significantly increasing the risk of delamination in composite laminates [15]. Additionally, CFRP laminates possess brittle interlaminar interfaces and are prone to delamination failure under out-of-plane loads [16], which exacerbates the structural safety and interlaminar damage issues arising from functional integration. Therefore, achieving the simultaneous optimization of electrothermal functionality and structural reliability has emerged as an urgent and critical scientific challenge in the design and application of structural–functional integrated composites.

Rational selection of materials and structural design of interlayers are crucial for the integrated optimization of both functionality and mechanical properties. As the core component of the interlayer, the electrothermal functional film must not only achieve high-efficiency electrothermal conversion but also exhibit excellent material compatibility and resin wettability to ensure the structural integrity of the final composite. Among the various electrothermal materials, carbon-based materials, particularly carbon nanotubes (CNTs), have garnered significant attention due to their high electrical conductivity, excellent thermal conductivity, and remarkable axial mechanical strength [17,18]. CNTs are typically utilized in macroscopic aggregated forms, such as powder, particles, and fiber veils. Given the structure-dependent performance of these materials, CNTs with a high aspect ratio and unidirectional alignment [19,20,21] offer superior advantages in electrothermal conversion. The macroscopically continuous and microscopically porous structure of CNTv ensures good processability and facilitates sufficient resin infiltration. This continuous CNTv architecture acts as a typical interleaf toughening phase: its macroscopically connected and aligned fiber network can induce crack deflection, fiber pull-out and fiber bridging in the interlaminar region of CFRP, which is defined as veil/interleaf toughening by continuous CNT architectures and achieves macroscopic interlaminar toughening through these extrinsic energy dissipation mechanisms. Owing to this structural feature and the excellent intrinsic mechanical properties of CNTv, our previous studies have demonstrated the potential of CNTv to enhance the interlaminar toughness of composites. In contrast to the macroscopic toughening of continuous CNTv, individual or small-bundled dispersed CNTs can realize matrix toughening by uniformly dispersing in the epoxy resin matrix: the dispersed CNTs participate in load transfer during the deformation of the resin matrix and exert a pinning-bridging effect during crack propagation to consume fracture energy, thus intrinsically improving the strength and toughness of the polymer matrix itself. Fu et al. [22] systematically investigated the effects of the resin infiltration state and film thickness on the interlaminar fracture behavior of CFRP interleaved with CNTv, revealing a toughening mechanism dominated by crack deflection and bridging. Experiments revealed that a 15 μm thick CNTv interlayer achieved the maximum Mode II toughening, elevating the G_IIC_ by ~67.58%. Consequently, CNTv is a promising embedded electrothermal element, capable of providing heating functionality while significantly improving the fracture resistance of laminates.

Furthermore, insulating layers were arranged around the electrothermal layer to isolate the carbon fibers and form a stable circuit in the interlaminar region. The material selection and structural design of insulating layers must also satisfy multiple requirements, including excellent electrical insulation, good resin wettability, and high intrinsic mechanical strength [23]. Among the numerous insulating materials, GF possesses excellent electrical insulation [24] and is thus the preferred insulating material for electrothermal composite systems. Compared with commonly used dense GF mesh fabric, ultra-thin GF mesh fabric is more conducive to resin infiltration and integral compounding. Existing studies [25,26,27] have shown that this type of structure has the potential to trigger toughening mechanisms, such as fiber bridging, plastic deformation, and crack deflection in the interlaminar region of composites, thereby improving their delamination resistance. Therefore, the use of ultra-thin GF mesh fabric as insulating interlayers is expected to achieve reliable electrical isolation and simultaneous optimization of interlaminar toughening.

Building upon our prior research, CNTs were incorporated into the electrothermal element as a modifying phase to tailor the interfacial and electrical characteristics. CNTs are uniformly dispersed within the interstice resin of CNTv through a pre-impregnation process. The combination of dispersed CNTs and continuous CNTv constructs a multi-scale synergistic toughening system for the interlayer structure: the dispersed CNTs optimize the interfacial bonding between the continuous CNTv and the epoxy matrix, as well as between CNTv and the glass fiber mesh fabric, via a nanoscale pinning effect, while the continuous CNTv provides a macroscopic toughening framework for the interlayer. This multi-scale collaboration realizes the synergistic enhancement of interlaminar toughness from the nano-scale (matrix toughening) to the micro/macro-scale (veil interleaf toughening), which is the core of the multiscale synergistic effects from combining dispersed CNTs and continuous CNT architectures. This approach facilitates the construction of a cross-scale three-dimensional conductive network, thereby optimizing the electrothermal response characteristics of the electrothermal layer [28]. Additionally, CNTs are anticipated to enhance interfacial bonding at the nanoscale via the pinning and bridging effect, inhibit crack propagation, and consequently improve interlaminar toughness synergistically [29]. Ultimately, based on the aforementioned material selection and structural design, a functional interlayer with a multi-layer composite structure of GF/CNTs-CNTv/GF was developed. It is expected that the simultaneous optimization effect of multi-component and multi-scale structures will maintain or even enhance the delamination resistance of CFRP laminates while ensuring high-efficiency and stable electrothermal conversion.

Currently, studies on the mechanisms governing G_IIC_ of multi-component and multi-scale interlayer systems (as exemplified by the GF/CNTs-CNTv/GF structure) in CFRP laminates remain scarce [30]. Although this structural design effectively integrates functions such as electrothermal heating, the complexity of its material system and configuration presents significant challenges to its mechanical properties, particularly interlaminar delamination resistance [31]. Therefore, this study focuses on interlaminar fracture toughness, a critical mechanical performance indicator of composites, to systematically investigate the fracture behavior of GF/CNTs-CNTv/GF functionalized CFRP under Mode II loading and elucidate their multi-scale simultaneous optimization toughening mechanism. By regulating key variables such as interlayer configuration and CNTs loading content, the G_IIC_ values of the laminates were evaluated via end-notched flexure test [32]. Furthermore, fracture morphologies and crack propagation paths were analyzed in depth using multi-scale characterization techniques, including SEM and ultra-depth-of-field three-dimensional microscopy. This study not only provides essential data and a theoretical basis for evaluating the mechanical reliability of such multifunctional composites but also offers novel design concepts and practical strategies for developing a new generation of CFRP composites with both high functionality and excellent interlaminar fracture toughness.

## 2. Experimentation

### 2.1. Materials

T-700 grade carbon fiber (CF) unidirectional prepreg (WP-R1360 epoxy resin system) provided by Huibai Advanced Materials Co., Ltd. (Shanghai, China), with a single-ply thickness of approximately 0.125 mm. The fiber volume fraction of the prepared CF laminate was approximately 70 vol%, and the resin content of the prepreg was about 30 wt%. The interlaminar toughening phase comprised aligned CNTv, acquired from Shenzhen Xivan Technology Co., Ltd. (Shenzhen, China), with a thickness of approximately 10 μm. The nano-reinforcement phase consisted of CNTs with a diameter of 30–50 nm, a length of 0.5–2 μm, and a purity exceeding 98%. Auxiliary materials included a 60 μm thick GF mesh fabric, analytical grade acetone, and polyetheramine D-230 curing agent (Aladdin, Shanghai, China). The fabrication process utilized an ultrasonic cell disruptor (900 W; Shanghai, China) and a 0.3 t flat vulcanizing press (Huzhou, China).

### 2.2. Modification of CNT Veil Toughening Layer

#### 2.2.1. Solvent-Assisted Pre-Impregnation Process

To enhance the wettability of the “dry” CNTv, a stepwise impregnation process was implemented as a pre-treatment. Initially, the WP-R1360 epoxy resin was diluted with acetone at a mass ratio of 1:2 (*m*/*m*). Subsequently, polyetheramine D-230 was incorporated at an epoxy resin-to-hardener mass ratio of 100:35.2 (*m*/*m*). The mixture was stirred at a speed of 300 rpm for 5 min to achieve preliminary mixing before dispersion. The mixture was subjected to dispersion for 20 min using an ultrasonic cell disruptor (900 W) at a temperature of 25 ± 2 °C to achieve a homogeneous pre-impregnation solution. Thereafter, the CNTv were completely immersed in the pre-impregnation solutions and maintained at room temperature for 15 min to ensure comprehensive impregnation. Following removal, the fibers were allowed to air-dry at room temperature for 10 min to facilitate solvent evaporation. Subsequently, the CNTv were further dried in a vacuum drying oven at 40 °C for 5 min to ensure complete evaporation of the residual solvent.

#### 2.2.2. CNT Veil Modification

To improve interlaminar toughness and interfacial properties, pre-impregnation solutions with CNTs mass fractions of 0.1 wt%, 0.5 wt%, 1.0 wt%, 1.5 wt%, and 2.0 wt% were prepared individually. The mass fraction of CNTs is defined relative to the total mass of the epoxy resin-polyetheramine D-230 curing agent mixture in the pre-impregnation solution (excluding acetone). Following ultrasonic dispersion (900 W, 20 min) to achieve stable suspensions, the CNTv was impregnated according to the procedure outlined in Section 2.2.1, resulting in a series of CNTv toughening layers modified with CNTs.

For systematic description, the composite samples in this study were designated according to the “CGF-key variable” rule. The toughening layers prepared in this phase correspond to the final samples named: CGF-P (pre-impregnation CNTv without CNTs, CGF-0.0, hereafter referred to as CGF-0.0 for consistency), CGF-0.1, CGF-0.5, CGF-1.0, CGF-1.5, and CGF-2.0 (the number indicates the CNTs mass fraction in the suspensions). A control sample with an untreated as-received CNTv toughening layer was also prepared (CGF-N).

### 2.3. Preparation of Composites

Unidirectional CF prepreg were sectioned into samples measuring 25 cm × 15 cm for testing purposes. A laminate stacking configuration of ([0]_32_) was employed for laminate fabrication. The pre-impregnated CNTv toughening layer was strategically positioned between the 15th and 16th plies, flanked by GF mesh fabric layers above and below to establish the interlaminar structure. Polytetrafluoroethylene (PTFE) film was inserted at the interface between the CNTv interlayer and the adjacent GF mesh fabric to function as a precrack initiator. Following layup, a vacuum bagging process (vacuum level: 0.95 bar, degassing for 30 min) was utilized to eliminate interlayer air bubbles. Curing was executed in a flat vulcanizing press using a stepwise temperature protocol: pre-curing at 80 °C under pressure for 30 min, followed by a temperature increase to 125 °C for 90 min hold under pressure for complete curing. A constant pressure of 0.3 t was maintained throughout, resulting in laminates with a uniform thickness of approximately 3.2 mm.

Based on the aforementioned fabrication process and the various interlaminar designs, the following samples were systematically prepared and characterized. Their naming conventions and specific meanings are as follows. C0: Baseline CFRP without an interlayer. CG: Sample with only a pure GF mesh fabric interlayer. CGF-N: Sample with a GF/CNTv/GF interlayer in which the CNTv was unmodified. CGF-P: Sample containing a GF/CNTv/GF interlayer with pre-impregnated CNTv. CGF-0.1 to CGF-2.0: Samples with GF/CNTs-CNTv/GF interlayers containing varying CNTs mass fractions (0.1–2.0 wt%).

### 2.4. Characterization

#### 2.4.1. Interlaminar Fracture Toughness Testing

The mode II interlaminar fracture toughness was assessed in accordance with the ASTM D7905 standard [33] utilizing the End-Notched Flexure test. The final sample dimensions were 250 mm × 21 mm × 3.2 mm, with an initial pre-crack length (a_0_) of 30 mm.

The experiments were conducted using a universal testing machine. The support span (2L) was configured to 100 mm, and the loading rate was maintained at 0.5 mm/min. The critical load (P) was derived from the recorded load–displacement curve. The G_IIC_ value was computed using the following equation:

$$G_{IIc}=\frac{9a^{2}P\delta }{2w(2L^{3}+3a^{3})}$$

where δ represents the displacement at the critical load, a denotes the crack length, w is the sample width, and L is the half-span length. A minimum of five valid samples were tested under each condition, and the results were averaged. For each batch of composite samples with the same interlayer design, at least eight valid samples were prepared in one batch. A minimum of five valid samples were tested under each condition, and the results were averaged.

#### 2.4.2. Other Characterizations

To comprehensively examine the mechanisms of toughening and modes of failure, a multiscale characterization approach was employed utilizing SEM (Hitachi Regulus 8320, Hitachi High-Tech, Japan) and ultra-depth-of-field three-dimensional microscopy (VHX-7000, Keyence, Osaka, Japan). SEM was utilized to observe the micro-morphology of the CNTv in both their as-received and pre-impregnation states, along with the fracture surfaces of the End-Notched Flexure tested samples. Ultra-depth-of-field three-dimensional microscopy facilitated the observation of the crack propagation path in ENF samples, elucidating the influence of the interlayer structure on the damage modes of the laminate.

## 3. Result and Discussion

### 3.1. Design, Fabrication, and Microstructural Characterization

**Figure 1:**
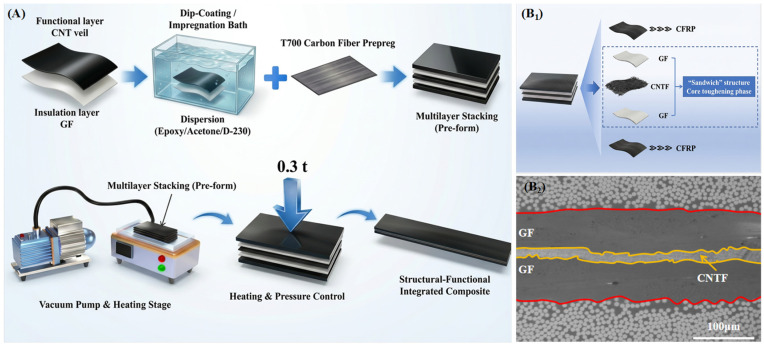
Schematic representation of the fabrication process and sandwich structure interlayer for interlaminar-toughened CFRP composites. (**A**) Flowchart of the fabrication process; (**B_1_**) Schematic illustration of the composite structure; (**B_2_**) Cross-sectional view of the toughening sandwich structure (yellow area: CNTv central toughening layer; red area: GF mesh fabric layer).

**Figure 2:**
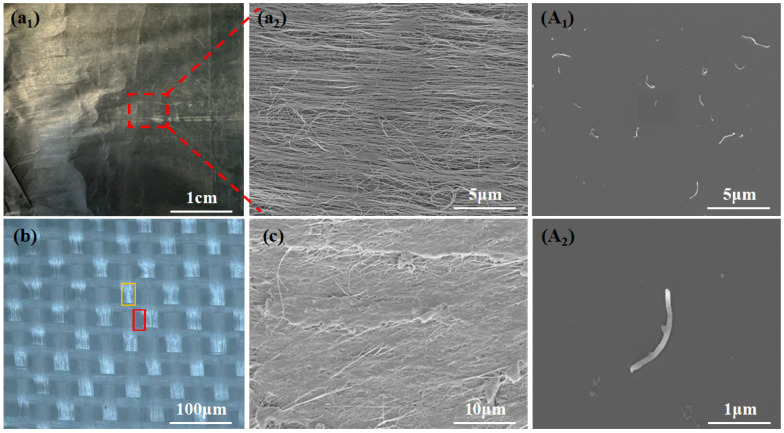
Characterization of the macro- and micro-morphology of essential raw materials. (**a_1_**) Macrograph of CNTv; (**a_2_**) SEM image of CNTv illustrating its highly aligned bundle structure; (**b**) Ultra-depth-of-field three-dimensional microscopy image of GF mesh fabric (red box: GF mesh fabric gap; yellow box: GF mesh fabric); (**c**) Cross-sectional SEM image of the CGF-P; (**A_1_**,**A_2_**) SEM image of the CNTs utilized.

In order to achieve a balanced integration of electrothermal heating performance and interlaminar fracture toughness in composite materials, this study has designed and introduced a “GF/CNTs-CNTv/GF” sandwich structure at the composite interlayer (Figure 1(B_1_,B_2_)). The core component of this structure is the CNTv, which provides electrothermal heating functionality. As depicted in Figure 2(a_1_,a_2_), the fibers of CNTv have diameters ranging from 20 to 50 nm and are highly aligned along the 0° direction. The CNTv, with a thickness of approximately 10 μm, exhibits a porous network structure conducive to resin infiltration and composite formation. It possesses excellent mechanical strength, high electrical conductivity, and high thermal conductivity [34], rendering it an ideal interlayer material for potentially activating both electrothermal heating and interlaminar toughening functions simultaneously [35]. However, the CNTv contains inherent internal fiber voids with dimensions on the order of nanometers. Therefore, achieving sufficient resin impregnation of the CNTv is a key challenge crucial for ensuring the structural integrity of the interlayer region. To address this issue, this study proposes a solvent-assisted pre-impregnation process (Figure 1A) for CNTv pre-treatment. The solvent reduces the viscosity and surface tension of the resin, facilitating its thorough penetration into the CNTv [36]. The cross-sectional SEM image of the CGF-P (Figure 2c) demonstrates that the epoxy resin fully encapsulated the CNTv, forming a dense CNTv/epoxy composite layer. GF mesh fabric layers were placed above and below the CNTv. The GF mesh fabric provides good electrical insulation and offers circuit protection for the central functional layer. This multilevel structural design, from fiber to mesh assembly (with a thickness of approximately 60 μm, Figure 2b), ensures resin impregnation and has the potential to activate interlaminar toughening mechanisms such as crack deflection, fiber bridging, and fiber fracture [23].

To further optimize the electrothermal heating performance and toughening effect of the interlayer, CNTs were introduced into the inter-fiber spaces of the CNTv layer via the solvent-assisted pre-impregnation method. To ensure the penetration and integration of CNTs within the CNTv interior, short CNTs with lengths of only 0.5–2 μm and diameters of 30–50 nm were selected. As nanofillers with excellent mechanical, electrical, and thermal properties [37], they are expected to increase the density of the conductive network, thus enhancing the electrical conductivity of the heating layer. And they are anticipated to contribute to matrix toughening and exert a mechanical interlocking effect, further enhancing the interfacial bonding between the phases within the complex sandwich structure [38].

### 3.2. Mode II Fracture Test

**Figure 3:**
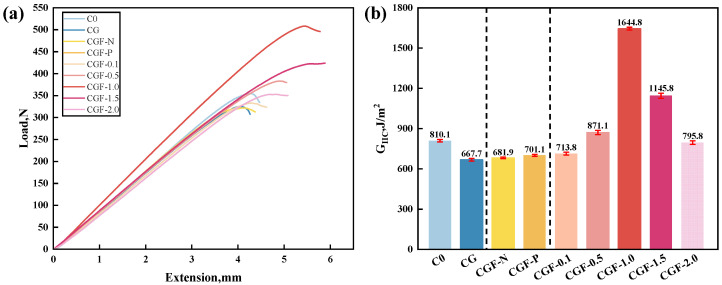
Results of Mode II interlaminar fracture toughness tests for CFRP with varying interlayer configurations. (**a**) Representative load–displacement curves derived from End-Notched Flexure testing; (**b**) Comparative analysis of G_IIC_ values.

To thoroughly examine the interlaminar fracture toughness of CFRP with sandwich-structured interlayer, baseline, single-interlayer, and CNT-modified/unmodified sandwich interlayer samples were prepared and subjected to the End-Notched Flexure test. The results are presented in Figure 3.

Initially, compared to the baseline sample (C0), the G_IIC_ value of CFRP with a GF mesh fabric (CG) decreased significantly, indicating that the single-component GF mesh fabric adversely affects the interlaminar properties of laminates. Building upon this structure, the CGF-N directly incorporated untreated CNTv, but its G_IIC_ showed no significant improvement. The sample with CNTv treated using the solvent-assisted pre-impregnation method (CGF-P) exhibited an increase in G_IIC_ compared to that of CGF-N. However, the improvement was marginal, indicating that the pre-impregnation treatment did not yield the anticipated improvement in interlaminar properties. Notably, the introduction of CNTs significantly enhanced the toughening effect of the sandwich interlayer. This effect varied with the CNTs content, exhibiting a trend of initial increase followed by a decrease. The corresponding sample achieved the optimal Mode II interlaminar fracture toughness when the CNTs content was increased to 1.0 wt%. At this concentration, the G_IIC_ value (1644.8 J/m^2^) increased by approximately 103.06% compared with the baseline C0 (810.1 J/m^2^), and by about 146.34% compared with the CG (667.7 J/m^2^), clearly demonstrating the remarkable toughening effect induced by CNTs modification. Subsequently, with further increases in CNTs content, G_IIC_ dropped to 1145.8 J/m^2^ (1.5 wt%) and 795.8 J/m^2^ (2.0 wt%), representing decreases of approximately 30.3% and 51.6% relative to CG-1.0. This indicates that within an ideal concentration range, the introduction of CNTs plays a crucial role in promoting the toughening effect of the sandwich interlayer, whereas excessive addition has a negative impact.

### 3.3. Mechanism Analysis

**Figure 4:**
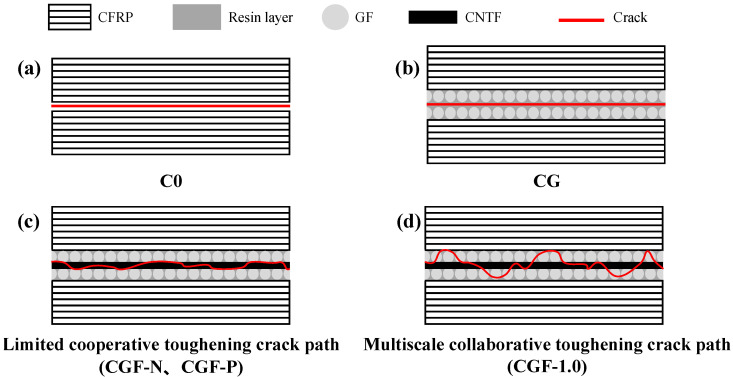
Schematic representations of crack propagation paths and toughening mechanisms for various interlaminar toughening systems. (**a**) Brittle interfacial debonding trajectory in the baseline C0 without an interlayer. (**b**) Smooth interfacial debonding trajectory in CG with only GF mesh fabric interlayers. (**c**) Crack propagation trajectory in interleaved samples utilizing untreated and pre-impregnation CNTv (CGF-N, CGF-P). (**d**) Tortuous crack trajectory in CGF-1.0, which was modified with CNTs.

To achieve a comprehensive understanding of the relationship between interlayer structure and interlaminar fracture toughness, this study utilized SEM and ultra-depth-of-field three-dimensional microscopy to characterize the fracture morphology and crack propagation paths of samples. Figure 4 presents a summary of the typical crack propagation paths and interlaminar fracture mode induced by different interlayer designs, offering a clear framework for the detailed analysis in this section.

#### 3.3.1. Analysis of Delamination Failure Modes

**Figure 5:**
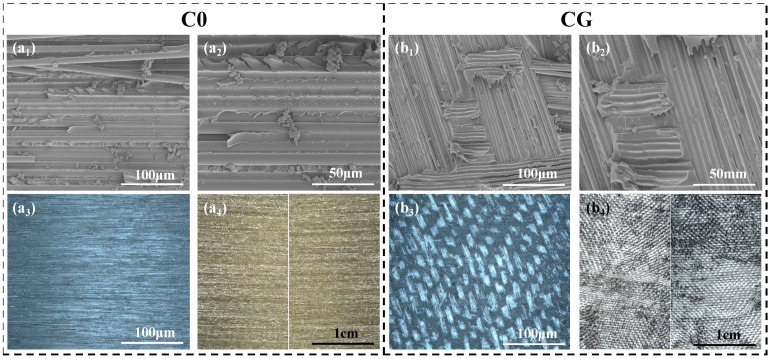
Analysis of the fracture surface morphology of both the baseline and GF mesh fabric interlayer CFRP laminates. (**a_1_**–**a_4_**) SEM images, ultra-depth-of-field three-dimensional microscopy image, and macrograph of the fracture surface for the baseline without interlayer; (**b_1_**–**b_4_**) SEM images, ultra-depth-of-field three-dimensional microscopy image, and macrograph of the fracture surface for CG with only a GF mesh fabric interlayer (bright regions in the ultra-depth-of-field three-dimensional microscopy image correspond to GF mesh fabric).

Initially, the delamination failure modes of various samples were examined. The baseline (C0) demonstrated typical brittle-fracture characteristics (Figure 4a). SEM images (Figure 5(a_1_,a_2_)) revealed a relatively flat and smooth fracture surface with a substantial amount of exposed carbon fibers. Continuous shear bands were observed in localized areas, indicating a failure mode predominantly governed by interfacial debonding between the carbon fibers and resin matrix, accompanied by limited cohesive resin failure. Characterization of the fracture surface morphology at the macroscopic scale (Figure 5(a_3_,a_4_)) further confirmed the features of scarce fiber bridging and a singular crack propagation path.

The fracture behavior of the CFRP altered significantly following the introduction of the GF mesh fabric interlayer (CG). Ultra-depth-of-field three-dimensional microscopy images and macrographs of the fracture surface (Figure 5(b_3_,b_4_)) indicated that the delamination surface of the CG was almost entirely covered with blocky bright spots, preliminarily identified as the exposed GF mesh fabric. This observation was further corroborated by the SEM images (Figure 5(b_1_,b_2_)), which showed that the delamination surface contained numerous GF, appearing smooth and clean. Resin-rich areas exhibited distinct traces of GF debonding with no evident shear deformation bands. This suggests that the primary delamination failure mode for CG is interfacial debonding between the GF mesh fabric and the epoxy resin-rich region (Figure 4b). In this interlayer system, the GF mesh fabric exerts a strong guiding effect on interlaminar crack propagation, failing to activate effective toughening mechanisms while simultaneously suppressing other energy-dissipation mechanisms. Consequently, this resulted in a G_IIC_ value lower than that of the baseline [39,40].

**Figure 6:**
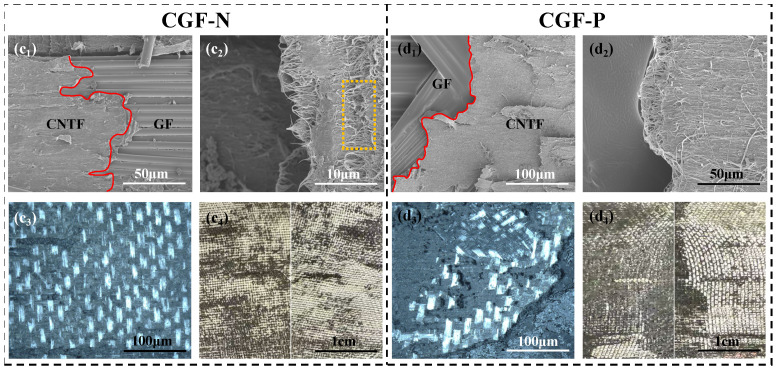
Characterization of the fracture surface morphology of CFRP laminates with CNTv interlayers. (**c_1_**–**c_4_**) SEM images, ultra-depth-of-field three-dimensional microscopy image, and macrograph of the fracture surface for sample CGF-N with untreated CNTv interlayer; (**d_1_**–**d_4_**) SEM images, ultra-depth-of-field three-dimensional microscopy image, and macrograph of the fracture surface for sample CGF-P with pre-impregnation CNTv interlayer (yellow dashed line: incompletely impregnated fiber bundles; red solid line: demarcation line between CNTv layer and GF mesh fabric layer; bright regions: GF mesh fabric; dark regions: CNTv).

Further investigation was conducted on the complex sandwich structure interlayer formed by incorporating a CNTv intermediate layer. Analysis of the delamination fracture surfaces of the CGF-N (without pre-impregnation CNTv) and CGF-P (with pre-impregnation CNTv) revealed similar morphologies. Both surfaces exhibited two distinct regions: a CNTv/resin composite zone and an exposed smooth GF (or GF/resin) layer beneath, due to the localized detachment of the CNTv composite from the GF mesh (Figure 6(c_1_–d_4_)). This observation indicates that under Mode II loading, delamination cracks in these two groups of samples propagated and deflected both within the CNTv layer and at the interface between the CNTv and GF mesh layers. Furthermore, in the CNTv/resin composite zones of the CGF-N fracture surface, local areas displayed “dry fiber tow” (Figure 6(c_1_–c_4_)), yellow dashed boxes), suggesting insufficient resin penetration and impregnation of the CNTv during interlayer formation. This introduced initial defects in the CNTv composite layer. In contrast, the fracture surface of the CGF-P (Figure 6(d_1_–d_4_)) shows that the CNTv was fully encapsulated by the epoxy resin, with no evident “dry fiber tow” phenomenon. This demonstrates that the solvent-assisted pre-impregnation process facilitated thorough resin infiltration and integration with the CNTv, improving the interfacial bonding between the CNTv and the resin, thereby enhancing the stress transfer capability within the CNTv composite layer [41].

In summary, the primary failure modes for both the CGF-N and CGF-P were intralayer tearing within the CNTv layer and weak interfacial debonding at the CNTv/GF interface. Although this process induced limited crack deflection and some CNTv fracture and pull-out, it failed to establish effective simultaneous optimization toughening. Their crack propagation paths can be categorized as the “Limited Simultaneous Optimization Effect” type, as illustrated in Figure 4c. Consequently, the Mode II interlaminar fracture toughness of both remained relatively low. Although CGF-P exhibited better structural integrity, it resulted in only a marginal improvement in the G_IIC_ value. Therefore, this interlayer design did not effectively overcome the failure mode dominated by weak interfacial debonding.

**Figure 7:**
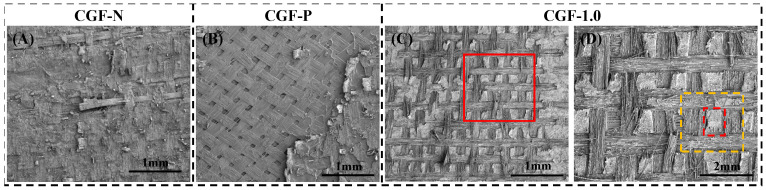
Analysis of fracture surface morphologies of CNTv-interlayered samples subjected to various treatments. (**A**) CGF-N with untreated CNTv; (**B**) CGF-P with pre-impregnation CNTv; (**C**) SEM image of CGF-1.0, which CNT veil modified using a 1.0 wt% CNTs dispersion, exhibiting a distinctive “mesh-block” fracture morphology; (**D**) Enlarged image of the area delineated by the red box in (**C**) (red dashed box: CNTv/resin composite area; yellow dashed box: GF mesh fabric/resin composite area).

Notably, the further modification of the CNTv layer through the incorporation of CNTs resulted in a substantial alteration of the delamination mode [42]. For instance, the fracture surface of the CGF-1.0 displays a distinctive “mesh-block” morphology. As illustrated in Figure 7C,D, the interior of the grid comprises square-shaped CNTv/resin composite zones with side lengths ranging from 100 to 200 μm, corresponding to the open areas within the GF mesh (Figure 2b). The edges consist of GF mesh fabric/resin composite regions or resin-rich zones, where the GF mesh have debonded and been extracted. This suggests that under Mode II loading, crack propagation in CGF-1.0 was impeded by the heterogeneous interlayer structure, resulting in periodic deflection both within the CNTv layer and at the interfaces above and below the GF mesh fabric layer.

In contrast to the delamination mode of samples without CNTs modification, which involved extensive peeling of weak interfaces (Figure 7A,B), the damage mode in this sample was more intricate, manifesting as intraphase delamination within the multiphase structure and periodic interphase deflections. This complexity is also a key factor in the successful activation of the toughening effect in this type of interlayer structure.

#### 3.3.2. Toughening Mechanisms of CNT-Modified Samples

As previously discussed, the samples modified with CNTs demonstrated a substantial enhancement in interlaminar fracture toughness, as well as distinctive delamination behaviors. Moreover, the toughening effect exhibited significant variation with the concentration of CNTs in the pre-impregnation solutions, with the G_IIC_ values initially increasing and subsequently decreasing (Figure 3). This observation suggests that the modification of pre-impregnation solutions through doping with CNTs is crucial for effectively activating the toughening effect of the sandwich interlayer structure. Consequently, this section is dedicated to analyzing the toughening mechanisms of the CNTs-modified samples.

**Figure 8:**
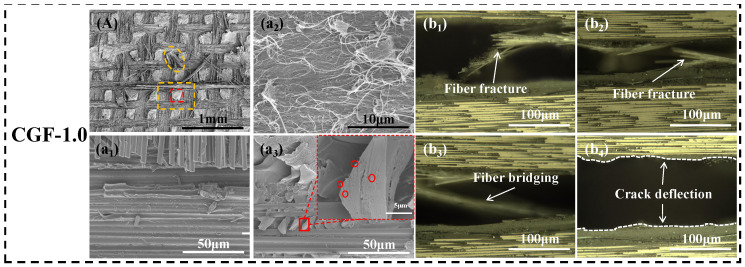
Analysis of the fracture morphology of the CGF-1.0,which CNT veil modified using a 1.0 wt% CNTs dispersion. (**A**) SEM image of the cross-sectional morphology (red dashed box: CNTv/epoxy composite zone; yellow dashed box: GF mesh fabric reinforced zone). (**a_1_**–**a_3_**) SEM images of the corresponding regions marked in (**A**), showing microscopic features: (**a_1_**) evident GF mesh fabric fracture; (**a_2_**) well-impregnated CNTv; and (**a_3_**) uniformly dispersed carbon nanotubes (red circles). (**b_1_**–**b_4_**) Ultra-depth-of-field three-dimensional microscopy images, demonstrating typical toughening behaviors including fiber fracture (**b_1_**,**b_2_**), fiber bridging (**b_3_**), and crack path deflection at heterogeneous phase interfaces (**b_4_**).

The sample demonstrating the most significant toughening effect, CGF-1.0, exhibited a distinctive “mesh-block” morphology on its fracture surface (Figure 7C and Figure 8). Within its interlayer, the CNTs-modified CNTv layer and the GF mesh fabric layer were interwoven, creating a periodic, tightly integrated heterogeneous composite system. Under Mode II shear loading, the crack was impeded from propagating along a single interface. Instead, it was directed to undergo periodic deflection and branching at the GF mesh, the CNTv composite layer, and their interphase interfaces (Figure 8(b_1_–b_4_)). The corresponding crack propagation path, as depicted in Figure 4d, exhibits high tortuosity and periodicity. This significantly elongated crack path enhances energy dissipation during fracture. Simultaneously, the periodically deflected crack perturbed the constituent phases within the interlayer. In the GF mesh fabric composite zones (Figure 8, yellow dashed boxes), it induced extensive GF bridging and fracture (Figure 8(b_1_–b_3_)), along with significant shear deformation and cohesive failure of the surrounding resin. In the CNTv/epoxy composite zones (Figure 8, red dashed boxes), CNTv bundles fractured and were pulled out under shear, while distinct slip shear bands were observed within the resin matrix (Figure 8a_1_). This indicates that the failure mode of the material transitioned from being dominated by large-scale weak interface debonding to intralayer damage within the toughening layer, encompassing a composite failure mechanism that includes periodic crack deflection, fiber pull-out and fracture, as well as resin deformation and cohesive failure.

It is important to highlight that the matrix strengthening and toughening effects of CNTs, along with their “nano-anchoring” effect at phase interfaces, are crucial for activating the synergistic toughening mechanisms within this system [40]. As depicted in Figure 8a_3_, the CNTs were uniformly dispersed within the epoxy matrix, either as individual tubes or small bundles. They were observed among carbon nanotube fiber bundles, within the gaps of the GF mesh fabric, and in the interfacial transition regions between the two phases (Figure 7C,D). This suggests that their infiltration into critical locations of the sandwich structure was not significantly impeded by the “filtering effect” of the GF mesh during processing [43]. The uniform and adequate dispersion of the nanoparticles ensured their dual role in matrix strengthening and toughening, as well as multiphase-interface reinforcement. On one hand, uniformly dispersed CNTs enhanced the strength and toughness of the resin matrix, thereby improving its plastic deformation capability under shear loading (Figure 8a_3_). CNTs enhance the matrix by uniformly dispersing to carry load and by consuming fracture energy through a “pinning-bridging” effect during crack propagation, thus synergistically improving the matrix’s strength and toughness [44,45]. Conversely, CNTs function as “nano-anchoring,” significantly enhancing the interfacial bonding between GF mesh, carbon nanotube fiber bundles, and the resin matrix through mechanical interlocking [46]. Particularly at the GF/CNTv heterogeneous interface, they form a strong and tough nanoscale transition layer, thereby improving the interphase bonding.

Owing to the effective reinforcement of multiphase interfaces by CNTs, this sandwich structure operated as a well-integrated heterogeneous system capable of resisting delamination failure. This reinforcement compelled the crack to deflect into the material’s interior at the strengthened interfaces, resulting in a shift in the predominant fracture mode from “interfacial debonding” to “failure within heterogeneous phases.” Throughout this process, macro-scale crack deflection, mesoscale fiber pull-out and fracture, and nanoscale CNTs bridging and matrix plastic deformation were synergistically activated. This led to the formation of a multilevel energy dissipation mechanism, ultimately evidenced by the distinctive “mesh-block” delamination morphology and the substantial enhancement of Mode II interlaminar fracture toughness.

**Figure 9:**
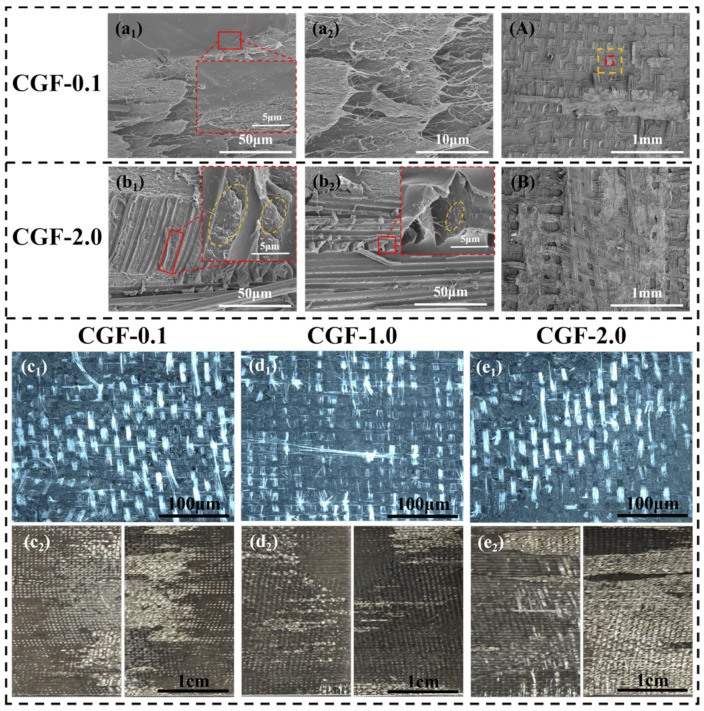
Analysis of the fracture morphology of modified samples with varying CNTs contents: (**a_1_**,**a_2_**,**A**) SEM images of the CGF-0.1 (in image (**A**), the red dashed box delineates the CNTv/epoxy composite zone, while the yellow dashed box delineates the GF mesh fabric reinforced zone); (**b_1_**,**b_2_**,**B**) SEM images of the CGF-2.0 (yellow curves: CNTs); (**c_1_**,**d_1_**,**e_1_**) ultra-depth-of-field three-dimensional microscopy images of the CGF-0.1, CGF-1.0, and CGF-2.0 (bright regions correspond to GF, and dark regions correspond to CNTv); (**c_2_**,**d_2_**,**e_2_**) macrographs of the fracture surfaces of the CGF-0.1, CGF-1.0, and CGF-2.0.

In contrast, when the concentration of CNTs in the pre-impregnation solution was low (0.1 wt%, CGF-0.1), preliminary “mesh-block” features emerged on the fracture surface (Figure 9(A,c_1_,c_2_)), indicating the initial formation of a heterogeneous structural framework. However, the insufficient CNTs content hindered the development of a sufficiently dense and uniform “nano-anchoring” network (Figure 9(a_1_,a_2_)). As a result, effective interfacial bonding and crack deflection were achieved only in localized regions. Consequently, mechanisms such as GF bridging and CNTv pull-out were not fully activated, and the multiscale simultaneous optimization toughening mechanism failed to fully develop and function, ultimately resulting in only a marginal improvement in the G_IIC_ value.

When the CNT content exceeded the optimal value (2.0 wt%, CGF-2.0), the “mesh-block” structure exhibited significant degradation or became indistinct (Figure 9(A,e_1_,e_2_)), indicating a shift in the delamination mode from periodic deflection between heterogeneous phases to propagation predominantly within the CNTv layer. The primary reason for this is the significantly increased viscosity of the pre-impregnation solution at high CNTs concentrations, which severely impedes uniform penetration and thorough impregnation within the CNTv bundles. This not only led to pronounced CNTs agglomeration within the CNTv layer (Figure 9(a_1_,a_2_)) but also resulted in poorly impregnated “dry fiber tow” defects in localized areas. The agglomerates act as stress concentrators, prone to inducing microcracks, whereas the impregnation defects become weak interfacial zones. Together, they provide preferential paths for crack initiation and propagation, thereby disrupting the effective control of crack behavior by the periodic heterogeneous structure, ultimately leading to a decrease in the toughening effect [47,48,49]. Thus, at high concentrations, CNTs no longer function as uniformly dispersed reinforcements and interface strengtheners but become detrimental factors in the form of agglomerates, compromising the structural homogeneity and interfacial integrity.

In summary, the toughening effect of CNTs was significantly dependent on their concentration. At a low content (0.1 wt%), insufficient interfacial strengthening prevents the full activation of simultaneous optimization toughening mechanisms. At a high content (2.0 wt%), CNTs agglomeration introduced defects that impaired the interfacial integrity and weakened the simultaneous optimization effect. Neither scenario allows the construction of a complete “nano-anchoring” network. Consequently, their crack propagation paths (Figure 4d) and toughening effects were far inferior to those of CGF-1.0. Only at the optimal content (1.0 wt%) can CNTs achieve uniform dispersion and fully exert their matrix strengthening/toughening and “nano-anchoring” effects, enhancing the interfacial bonding between the GF and CNTv/resin composite layer. This forces cracks to undergo periodic deflection and branching within the multiphase structure, synergistically activating various toughening mechanisms, including fiber bridging, fracture, pull-out, resin plastic deformation, and cohesive failure, ultimately leading to a significant enhancement in the Mode II interlaminar fracture toughness.

## 4. Conclusions

This study aimed to achieve a structurally and functionally integrated design for CFRP and to gain a comprehensive understanding of the interlaminar fracture toughness of complex, multiphase interlayer systems. To this end, a representative “GF/CNTs-CNTv/GF” sandwich interlayer structure was constructed and introduced into the interlaminar region of CFRP. The Mode II interlaminar fracture behavior of the composites was subsequently investigated, leading to the following conclusions.

The results demonstrate that effective simultaneous optimization toughening in complex interlayer architectures critically depends on the quality of enhanced interfacial bonding. In the absence of CNT modification, interlayered samples predominantly failed through interfacial delamination, characterized by extensive debonding at weak interfaces, which resulted in relatively low (or even inferior) interlaminar fracture toughness compared with the baseline composite. By contrast, introducing an appropriate amount of CNTs into the resin of the CNTv layer via a solvent-assisted pre-impregnation process markedly enhanced interfacial bonding. Under Mode II loading, this improvement triggered a fundamental change in the delamination behavior, yielding a distinctive “mesh-block” fracture-surface morphology. This morphology indicates that crack propagation was repeatedly disrupted and deflected within the heterogeneous interlayer, both inside the CNTv layer and at the interfaces adjacent to the GF mesh fabric layers. As a consequence, multiscale and multiphase simultaneous optimization toughening mechanisms were fully activated. The discontinuous GF mesh fabric and CNTv layer interlocked to form a tightly integrated heterogeneous structure, inducing periodic crack deflection within the interlayer. This process promoted extensive GF and CNTv pull-out and fracture, accompanied by pronounced resin cohesive failure and shear deformation. At the nanoscale, additional energy dissipation arose from CNTs pull-out and crack-tip bridging. Owing to these combined mechanisms, the CNTs-modified interlayer achieved a maximum Mode II interlaminar fracture toughness (G_IIC_) of 1644.8 J/m^2^, corresponding to an improvement of approximately 103.06% relative to the baseline laminate. This enhancement is attributed to the uniform dispersion of CNTs, which reinforced the resin matrix through effective load transfer and a synergistic pinning-bridging effect.

Moreover, the interlaminar fracture toughness exhibited a non-monotonic dependence on CNT concentration, increasing initially and then decreasing at higher loadings. Excessive CNT content significantly increased the viscosity of the pre-impregnation solution, leading to nanoparticle agglomeration and incomplete resin infiltration within the CNTv layer. The resulting internal defects and external nano-agglomerates acted as stress concentrators, degraded interfacial bonding, and suppressed simultaneous optimization toughening, ultimately causing a reduction in G_IIC_.

Overall, this work demonstrates that interfacial nano-engineering can fundamentally shift the Mode II interlaminar fracture behavior of CFRP from interfacial debonding-dominated failure to failure governed by heterogeneous phase interactions. The findings confirm the effectiveness of the GF/CNTs-CNTv/GF sandwich-structured interlayer in enhancing interlaminar fracture toughness and provide valuable theoretical insight and experimental guidance for the development of advanced multifunctional composite materials with superior damage tolerance.

## Data Availability

The data presented in this study are available on request from the corresponding authors.
